# Cyclooxgenase-2 Inhibiting Perfluoropoly (Ethylene Glycol) Ether Theranostic Nanoemulsions—*In Vitro* Study

**DOI:** 10.1371/journal.pone.0055802

**Published:** 2013-02-07

**Authors:** Sravan Kumar Patel, Yang Zhang, John A. Pollock, Jelena M. Janjic

**Affiliations:** 1 Graduate School of Pharmaceutical Sciences, Mylan School of Pharmacy, Duquesne University, Pittsburgh, Pennsylvania, United States of America; 2 Department of Biological Sciences, Bayer School of Natural and Environmental Sciences, Duquesne University, Pittsburgh, Pennsylvania, United States of America; Shanghai Jiao Tong University School of Medicine, China

## Abstract

Cylcooxgenase-2 (COX-2) expressing macrophages, constituting a major portion of tumor mass, are involved in several pro-tumorigenic mechanisms. In addition, macrophages are actively recruited by the tumor and represent a viable target for anticancer therapy. COX-2 specific inhibitor, celecoxib, apart from its anticancer properties was shown to switch macrophage phenotype from tumor promoting to tumor suppressing. Celecoxib has low aqueous solubility, which may limit its tumor inhibiting effect. As opposed to oral administration, we propose that maximum anticancer effect may be achieved by nanoemulsion mediated intravenous delivery. Here we report multifunctional celecoxib nanoemulsions that can be imaged by both near-infrared fluorescence (NIRF) and ^19^F magnetic resonance. Celecoxib loaded nanoemulsions showed a dose dependent uptake in mouse macrophages as measured by ^19^F NMR and NIRF signal intensities of labeled cells. Dramatic inhibition of intracellular COX-2 enzyme was observed in activated macrophages upon nanoemulsion uptake. COX-2 enzyme inhibition was statistically equivalent between free drug and drug loaded nanoemulsion. However, nanoemulsion mediated drug delivery may be advantageous, helping to avoid systemic exposure to celecoxib and related side effects. Dual molecular imaging signatures of the presented nanoemulsions allow for future *in vivo* monitoring of the labeled macrophages and may help in examining the role of macrophage COX-2 inhibition in inflammation-cancer interactions. These features strongly support the future use of the presented nanoemulsions as anti-COX-2 theranostic nanomedicine with possible anticancer applications.

## Introduction

Inflammation processes are involved in all stages of cancer development [1]. The tumor environment contains a wide variety of inflammatory cells such as mast cells, dendritic cells, natural killer cells and macrophages [2]. Macrophages, constituting up to 50% of tumor mass, are actively recruited during cancer development and play an important role in tumor angiogenesis and metastasis [3]. Cyclooxygenase-2 (COX-2) is an inducible pro-inflammatory enzyme implicated in tumor development and progression [4]. Recruitment of COX-2 expressing macrophages can create an inflammatory environment that strongly promotes tumor growth and angiogenesis [5]. COX-2 is involved in the synthesis of prostaglandin E_2_ (PGE_2_) which is necessary for the development of immunosuppressive cells (tumor associated suppressive macrophages and myeloid-derived suppressor cells) [6]. Therefore, we hypothesize that inhibiting COX-2 in tumor recruited macrophages can be a viable anticancer strategy.

Celecoxib, a COX-2 selective inhibitor is reported to reduce cancer risk and suppress tumor growth in preclinical and clinical studies [4], [7]–[9]. It acts as a multifunctional drug that simultaneously induces COX-2 independent apoptosis, inhibits PGE_2_ mediated anti-apoptotic proteins and inhibits angiogenesis [10]. Recently, celecoxib has shown to alter the phenotype of macrophages from protumor (M2) to antitumor (M1) subtype via COX-2 inhibition [11]. However, celecoxib, classified as a BCS (Biopharmaceutics classification system) class II drug, has very poor aqueous solubility of 7 µg/mL [12] and 22–40% oral bioavailability in dogs [13] (to our knowledge absolute bioavailability in humans has not been reported). Celecoxib is also rapidly eliminated from the plasma further lowering drug levels at the tumor site [14], [15]. In clinical cancer studies, celecoxib is administered orally at high doses (200–400 mg, twice daily) for several months leading to cardiovascular side effects, which may be severe [16]. To overcome these limitations, nanoparticle formulation of celecoxib was recently reported for colon cancer treatment in a human xenograft mouse model [15]. Based on these findings, we propose that the celecoxib loaded theranostic nanomedicine can suppress COX-2 activity in the circulating macrophages and allow us to track the macrophages tumor infiltration dynamics by molecular imaging (^19^F magnetic resonance and near-infrared fluorescence).

Integration of diagnosis with therapy (theranostics) in a single nanocarrier could facilitate visualization of nanocarrier biodistribution and treatment response. This ultimately enables assessment of safety, toxicity and efficacy of the therapeutic intervention [17] leading to personalized medicine. Multiple imaging approaches are being investigated for this purpose such as: using optical probes, radioactive ligands, magnetic resonance imaging (MRI) and ultrasound contrast agents [18]–[21]. Near-infrared fluorescence (NIRF) imaging is a promising technique due to low near-infrared (NIR) absorbance by living tissues, high detection sensitivity and minimal autofluorescence [22]–[24]. However, *in vivo* NIRF imaging is semi-quantitative with limited tissue penetration [25]. ^19^F MRI has unlimited tissue penetration and is a quantitative technique [26], [27]. ^19^F MRI is widely used to track the *in vivo* behavior of *ex vivo* perfluorocarbon (PFC) labeled cells [28], [29]. ^19^F magnetic resonance (MR) signal provides *in vivo* localization of exogenously introduced PFCs while conventional ^1^H MRI provides the anatomical context [29]–[31]. However, for effective imaging with ^19^F MRI, relatively large amounts of ^19^F nuclei (minimum of 7.5×10^16^ atoms per voxel) at the target site is required in preclinical models [27]. By coupling NIRF and ^19^F MR imaging modalities, sensitivity, specificity and high tissue penetration can be obtained [24].

Aspects of dual mode imaging of nanoemulsion have been previously reported [19], [24]. ^1^H MRI contrast agents in combination with NIRF imaging agents have been used as theranostic nanomedicine [19]. We recently reported a tyramide conjugated PFPE nanoemulsion with dual mode imaging capabilities [32]. In recent studies, macrophages were labeled *in vivo* by intravenously (i.v.) injected PFC nanoemulsions and their migration to the inflammation sites was monitored by ^19^F MRI [33], [34].

Here, we report for the first time theranostic nanomedicine integrating ^19^F MRI and NIRF imaging agents for simultaneous drug delivery and macrophage tracking. The presented theranostic PFC nanoemulsion design is innovative in that: 1) It incorporates a selective COX-2 inhibitor; 2) It can serve as a multimodal biological probe for studying the role of COX-2 in macrophage-tumor interaction; and 3) Can be imaged by two complimentary molecular imaging techniques-NIRF and ^19^F MR. Achieving the balance between imaging (^19^F MRI and NIRF) and therapeutic functionalities (COX-2 inhibition) in a single nanocarrier is critical. Using ^19^F NMR labeling, NIRF signal and COX-2 inhibition we achieved this balance successfully in *in vitro* cell culture studies. Targeting COX-2 in macrophages with a dual mode theranostic (^19^F MRI/NIRF capabilities) is shown for the first time. We report detailed *in vitro* characterization and *ex vivo* biological testing of the PFPE theranostic nanoemulsion in mouse macrophages.

## Materials and Methods

### Materials

Celecoxib was purchased from LC Laboratories® (Woburn, MA, USA). Miglyol 810N was generously donated by Croda® International Plc. Pluronic® P105 was obtained from BASF Corporation. Cremophor® EL was purchased from Sigma-Aldrich. Perfluoropoly (ethylene glycol) ether (produced by Exfluor Research Corp., Roundrock, TX, USA) was generously provided by Celsense Inc., Pittsburgh, PA, USA and used without further purification. CellVue® NIR815 (786 nm/814 nm) and CellVue® Burgundy (683 nm/707 nm) Fluorescent Cell Linker Kit was purchased from Molecular Targeting Technologies, Inc. (MTTI), West Chester, PA, USA. 0.4% Trypan blue solution was obtained from Sigma-Aldrich. CellTiter-Glo® Luminescent Cell Viability Assay was obtained from Promega Corporation, WI, USA. Prostaglandin E_2_ enzyme-linked immunosorbent assay (ELISA) kit was purchased from Cayman Chemical Company, MI, USA. Adherent mouse macrophage cell line (RAW 264.7) was obtained from American Type Culture Collection (ATCC), Rockville, MD, USA and cultured according to the instructions. Dulbecco's modified eagle medium (DMEM; GIBCO-BRL, Rockville, MD, USA) for cell culture experiments was supplemented with 10% fetal bovine serum (FBS), Penicillin/Streptomycin (1%), L-Glutamine (1%), HEPES (2.5%) and 45% D(+) glucose (1%). Trypsin EDTA, 1× was obtained from Mediatech, Inc., VA, USA. All cells were maintained in 37°C incubator with 5% carbon dioxide. Purified mouse anti-mouse CD45.1 monoclonal antibody conjugated to FITC (fluorescein isothiocyanate) used for cell labeling was obtained from BD Pharmingen™, Material No. 553775. Antifade ProLong® Gold (Invitrogen) was used as the mounting medium. Lysotracker® Green DND-26 and Hoechst 33342 were obtained from Invitrogen.

### Preparation of PFPE nanoemulsions

PFPE nanoemulsions were prepared using a mixture of nonionic surfactants, Pluronic® P105 (P105) and Cremophor® EL (CrEL). A premade aqueous solution of mixed surfactants was used.

#### Preparation of CrEL/P105 surfactant mixture

A solution containing mixed surfactants was prepared as follows: P105 (4 g) was dissolved in 100 mL water by stirring slowly at room temperature for the final concentration of 4% w/v (weight/volume). CrEL 6% w/v in water was prepared by magnetic stirring at room temperature. The two solutions were gently mixed at room temperature in 1∶1 v/v (volume/volume) ratio in a 500 mL round bottomed flask. The flask was placed in a water bath preheated to 45°C and slowly rotated for 20 min. The solution was then chilled on ice for 15 min, and stored in the refrigerator until use. The final concentration of this mixed surfactant solution was 5% w/v, where 2% w/v was P105 and 3% w/v was CrEL.

#### General procedure for the preparation of nanoemulsions using microfluidization

PFPE formulations contained 1.38% w/v CrEL, 0.92% w/v P105, 7.24% w/v PFPE, 3.8% w/v Miglyol 810N, 0.02% w/v celecoxib, 0.24 µM NIRF dye (Cellvue® NIR815 or Burgundy) and deionized water (final volume to 25 mL). Celecoxib (5 mg) was first dissolved in 0.95 g of Miglyol 810N by overnight stirring while 6 µL of NIRF dye stock solution (1 mM in EtOH) was added before blending with PFPE. PFPE oil (1.81 g) was transferred to a 500 mL round bottomed flask containing celecoxib, NIRF dye and Miglyol 810N and stirred at 1200 rpm, room temperature for 15 min. To this 11.5 mL (0.575 g of mixed surfactant) of mixed surfactant solution was added and stirred at 1200 rpm for additional 15 min. To this mixture, 11.5 mL of deionized water was added and stirred under ice cold conditions for 5 min at 1200 rpm. The coarse emulsion was microfluidized on a Microfluidics M110S for 30 pulses under recirculation mode (inlet air pressure ∼80 psi; operating liquid pressure ∼17500 psi) and temperature was noted. The nanoemulsion was sterilized using sterile 0.22 µm cellulose filter (Millex® - GS, 33 mm). Filtered nanoemulsion samples (1.5 mL) were stored at 4°C and 25°C to assess the stability. The bulk of the nanoemulsion was stored at 4°C until use. Nanoemulsion without celecoxib and NIRF dye was prepared in the same way to serve as the control. Table 1 show components of all the nanoemulsions (**A**, **B** and **C**) formulated. PFPE used in the nanoemulsions is a clear liquid (d  = 1.81 g/mL) represented by the formula CF_3_O(CF_2_CF_2_O)_n_CF_3_, where n = 4–16, with the average molecular weight of 1380 g/mol.

**Table 1:** Composition of nanoemulsions.

Nanoemulsion Component	A mg/mL	B mg/mL	C^a^ mg/mL
Celecoxib	0	0.2	0.2
PFPE	72	72	72
Miglyol 810N	38	38	38
Cremophor® EL	13.8	13.8	13.8
Pluronic® P105	9.2	9.2	9.2
NIRF Dye	µM	µM	µM
Cellvue® NIR815	0	0.24	0
Cellvue® Burgundy	0	0	0.24

a Nanoemulsion **C** is used for confocal microscopy of labeled macrophages.

### Characterization

Nanoemulsions were characterized by dynamic light scattering (DLS) measurements (Zetasizer Nano, Malvern, UK), ^19^F NMR (nuclear magnetic resonance) (Bruker, 470 MHz) and NIRF imaging (Odyssey® Infrared Imaging System, LI-COR Biosciences, NE, USA).

#### Droplet size and zeta potential measurements by DLS

The size distribution of the nanoemulsion droplets in aqueous medium was determined by DLS using Zetasizer Nano. Measurements were taken after diluting the nanoemulsion in water (1∶39 v/v). Measurements were made at 25°C and 173° scattering angle with respect to the incident beam. The stability of nanoemulsions was assessed by measuring the hydrodynamic diameter (Z average) and half width of polydispersity index (PDIw/2) at different time points (days). The stability of nanoemulsions incubated (37°C, 5% CO_2_) in cell culture medium (DMEM with 10% FBS) for 24 h was tested under same conditions. Nanoemulsions were monitored by DLS at two storage temperatures, 4°C and 25°C. Zeta potential was measured at same dilution using specialized zeta cells with electrodes following the manufacturer instructions.

#### ^19^F NMR measurements of nanoemulsions

^19^F NMR was recorded on nanoemulsions (and dilutions in water) with trifluoroacetic acid (TFA) as the internal standard in borosilicate NMR tubes (5 mm diameter). Briefly, nanoemulsion and 0.02% v/v TFA in water solution were mixed in 1∶1 v/v ratio (200 µL each) and spectra recorded (Bruker, 470 MHz). ^19^F NMR peak around −91.5 ppm corresponding to 40 fluorine nuclei was integrated with TFA (set at -76.0 ppm) as reference. Amount of PFPE per mL nanoemulsion was quantified based on the number of ^19^F under PFPE peak at −91.5 ppm (see Equation S1 for calculation).

#### NIRF imaging of nanoemulsions

NIRF images of the above prepared NMR samples were recorded on Odyssey® Infrared Imaging System. Nanoemulsion **B** loaded with celecoxib and NIRF dye was imaged. The NMR tubes with nanoemulsions were aligned and carefully taped to a paper, placed in the sample compartment and imaged. Images at 785 nm excitation wavelength and emission above 810 nm were collected. Imaging parameters include an intensity setting of 2 and 2.5 mm focus offset. NIRF signal was quantified from the obtained images using the instrument software (Odyssey® Imager v.3). Nanoemulsion **A** was used to correct for the fluorescence background. The total area corresponding to the nanoemulsion (with aqueous TFA) in the NMR tube was carefully selected for quantification after setting the nanoemulsion **A** fluorescence as background in the instrument software.

#### Drug content in nanoemulsion

A validated high performance liquid chromatography (HPLC) method was used to assess celecoxib content in nanoemulsion **B**. A previously reported method was adopted [35] and required validation parameters such as specificity, linearity, accuracy, intra-day and inter-day precision, limit of quantification and limit of detection were evaluated for celecoxib. Reverse phase chromatography was performed using C18 column (Hypersil Gold C18 150 mm×4.6 mm, 5 µm pore size) and 75∶25 methanol-water combination. Analysis was performed at isocratic conditions with the flow rate of 1 mL/min at 25°C column temperature. The detection wavelength was 252 nm. Celecoxib showed a sharp peak at 3.8 min retention time. HPLC was calibrated in the concentration range of 0.15–20 µg/mL celecoxib (correlation coefficient R^2^>0.999). To assess drug content, nanoemulsion **B** (250 µL) was dissolved in 10 mL methanol and vigorously vortexed. The mixture was centrifuged at 4000 rpm (Centrifuge 5804 R, 15 amp version) for 10 min. Supernatant was collected and analyzed for celecoxib. Analysis was carried out in triplicates. All the formulation ingredients were analyzed separately for possible interference using same chromatographic conditions.

### Cell Culture

#### Cell viability

Cell viability was assessed using CellTiter-Glo® luminescence assay. Briefly, mouse macrophages (RAW 264.7) were plated in 96 well plate at 10,000 cells/well. After overnight incubation at 37°C and 5% CO_2_, culture medium was removed and adhered cells were exposed to nanoemulsions **A** and **B** (prediluted in complete medium) at different PFPE concentrations and incubated overnight. 50 µL of the medium was carefully removed and 25 µL of CellTiter-Glo® analyte was added to each well. The plate was shaken for 20 min at room temperature to induce cell lysis. 60 µL of the cell lysate was transferred to a white opaque 96 well plate and luminescence was recorded on Perkin Elmer Victor 2 Microplate Reader.

#### Cell labeling

To assess the *in vitro* behavior of the nanoemulsions, cell labeling studies were conducted on mouse macrophages. Cells were cultured in 6 well plates at 0.3 million per well for 48 h. After aspirating the medium, cultured cells were washed with medium and phosphate-buffered saline (PBS). Cells were exposed to celecoxib and NIRF dye loaded nanoemulsion **B** (prediluted in medium) with concentration of PFPE ranging from 0.09 to 1.4 mg/mL. 2 mL of nanoemulsion **B** containing medium was added to each well. Cells were incubated for 24 h at 37°C and 5% CO_2_. Cells were washed (2×) with complete medium to remove non-internalized nanoemulsion and detached using trypsin. Detached cells were collected and centrifuged at 1100 rpm for 5 min. The supernatant was removed and the cell pellet was resuspended in complete medium and counted using Neubauer hemocytometer. To count the cells, equal volume of cell suspension and 0.4% Trypan blue cell staining solution were mixed and 25 µL of this mixture was used for cell counting. Cells were centrifuged again at 2000 rpm for 10 min to ensure complete removal of non-internalized nanoemulsion. After removing the supernatant, 180 µL of deionized water and 200 µL of 0.02% v/v aqueous TFA solution was added to the cell pellet, vortexed and transferred to 5 mm borosilicate NMR tubes.

#### ^19^F NMR measurements of labeled cells

NMR tubes with the labeled cell lysate (∼0.4 mL) prepared as described above were subjected to ^19^F NMR analysis to quantify the total fluorine content in the cells. The number of ^19^F per cell (Fc) was calculated using the following formula Fc = [(Ic/Ir)Nr]/Nc [36] , where (Ic/Ir) is the ratio of the integrated values of the PFPE peak in the cell pellet around −91.5 ppm corresponding to 40 fluorine nuclei divided by the TFA reference peak at −76.0 ppm, Nr is the total number of ^19^F in the TFA reference sample and Nc is the total cell number in the pellet.

#### NIRF measurements of labeled cells

NMR tubes containing labeled cells, TFA and water were directly imaged in Odyssey® Infrared Imaging system. Briefly, the NMR tubes were aligned and carefully taped to a paper, placed in the sample compartment and imaged. Images at 785 nm excitation wavelength and emission above 810 nm were collected. Imaging parameters include an intensity setting of 8 and 2.5 mm focus offset. Images were quantified using the instrument software and unexposed cells were used for background correction.

#### Fluorescence microscopy

Images of nanoemulsion labeled mouse macrophages were captured using confocal microscopy (Leica TCS SP2 spectral confocal microscope, Leica Microsystems) to assess the intracellular distribution of the nanoemulsion. Macrophages were cultured for 24 h on glass cover slips (Fisherfinest, 22×22-1) placed in a 6-well plate at a concentration of 10^5^ cells per well. Cultured macrophages were exposed to nanoemulsion **C** (21 µL nanoemulsion/mL medium; 2 mL total) for 24 h. After removing 1 mL medium, cells were fixed in 1 mL of 4% paraformaldehyde for 30 min. The medium in the cultured confocal plates (with glass cover slips) was carefully removed and washed with PBS (supplemented with 1% FBS). A stock solution of FITC dye conjugated mouse antimouse CD45.1 antibody (CD45-FITC) in 1% FBS in PBS was prepared at 1 µg/mL concentration. Cells in each well were exposed to 1 mL of the stock solution and left undisturbed at room temperature. After 15 min, dye solution was removed and washed with 1% FBS in PBS twice. Each cover slip was transferred to a microscopy slide with antifade mounting medium (ProLong® Gold, Invitrogen). Images were captured on a spectral analyzer confocal microscope. For visualizing FITC, excitation was achieved with the blue Ar laser 488 nm and emission window of 500 nm to 590 nm. Visualizing the Cellvue® Burgundy dye was achieved with the red HeNe 633 nm laser excitation and emission window of 640 nm to 850 nm. A transmission DIC image is acquired simultaneous to each confocal scan.

#### PGE_2_ assay

To investigate the *in vitro* therapeutic efficacy of the drug carrier, effect of nanoemulsions on PGE_2_ production by macrophages was assessed. Efficacy of nanoemulsion as drug carrier was assessed by comparing the effect on PGE_2_ production with free drug. Cells were plated in 6 well plates at 0.3 million cells/well and incubated overnight. Cells were exposed to nanoemulsion **B** at 1.4 mg/mL PFPE concentration (9.28 µM celecoxib), free drug dissolved in DMSO (9.28 µM) and DMSO. Fresh medium was added to unexposed cells. After overnight incubation, all wells were washed (2×) with medium and PBS. Bacterial toxin lipopolysaccharide (LPS) at 1 µg/mL diluted in medium (2 mL total) was added to each well with cells (exposed and unexposed) and incubated. Unexposed cells treated with LPS were designated as control and unexposed cells without LPS activation were designated as untreated. After 4 h incubation, supernatant was collected and analyzed using commercially available PGE_2_ ELISA kit. Samples were analyzed at two different dilutions (1∶4 and 1∶9) and two replicates of each dilution were used. Assessment of PGE_2_ production in the supernatant and data analysis was performed according to the manufacturer instructions.

## Results and Discussion

A novel COX-2 inhibiting PFC theranostic nanoemulsion with dual imaging capabilities (NIRF and ^19^F MR) was prepared. Design, formulation and *in vitro* evaluation are discussed in detail.

### Theranostic PFPE nanoemulsion design

**Figure 1:**
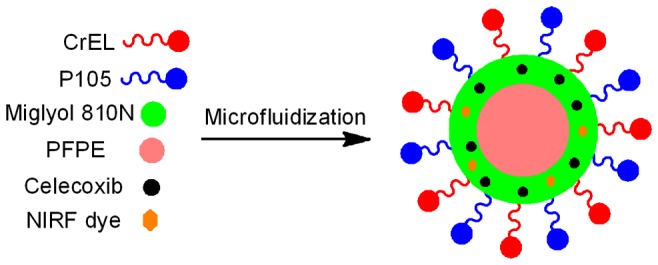
Proposed nanoemulsion droplet. Droplet carrying celecoxib, perfluoropoly (ethylene glycol) ether (PFPE) and near-infrared fluorescence (NIRF) dye. Cremophor EL® (CrEL) and Pluronic® P105 (P105) are the nonionic surfactants. Miglyol 810N is the hydrophobic oil phase.

Presented here is a novel PFC nanoemulsion designed to label macrophages upon exposure and inhibit their COX-2 activity. In this study, the PFC nanoemulsion has three key components (a) the anti-inflammatory drug celecoxib (b) NIRF dye for fluorescence imaging and (c) PFPE for ^19^F MRI. A proposed schematic of the nanoemulsion droplet is shown in Figure 1.

PFPE was chosen as the ^19^F imaging tracer to facilitate future *in vivo* imaging of the theranostic with ^19^F MRI and *ex vivo* ^19^F NMR cell loading quantification [29]. PFPE is desirable for *in vivo* ^19^F MRI due to the large number of magnetically equivalent fluorine nuclei. Further, this molecule shows high chemical and biological inertness. To date no metabolizing enzymes have been known to breakdown PFCs that can produce reactive intermediates [37]. PFPE shows a single main peak around −91.5 ppm [38] in the ^19^F NMR spectrum corresponding to the monomer repeats CF_2_CF_2_O. The total number of magnetically equivalent fluorines around −91.5 ppm is 40. A small peak around −59 ppm in the PFPE spectrum is not MRI detectable and hence its presence does not affect the image analysis [36]. PFPE was previously used for *in vivo* ^19^F MRI tracking of *ex vivo* labeled immune cells [29], [36] and is currently tested in cancer patients as immunotherapy imaging agent [39]. Due to high biological inertness, PFPE elimination is slow and relies on the reticuloendothelial system followed by expiration through lungs [37]. This is the general clearance profile for most PFCs used in biomedical applications [27], [37]. To enable intracellular fluorescence microscopy and future *in vivo* NIRF imaging of the theranostic, CellVue® NIR815 (excitation max = 786 nm, emission max = 814 nm) or Burgundy (excitation max = 683 nm, emission max = 707 nm) lipophilic dyes were selected. We incorporated two imaging agents to provide complimentary information about *in vivo* nanoemulsion accumulation by ^19^F MR and NIRF imaging modalities. Nanoemulsions can be imaged quantitatively in deep tissues using ^19^F MRI. NIRF imaging can enable visualization of nanoemulsion accumulation even at low amounts due to its sensitive nature.

Pure PFC as a major component of PFC nanoemulsions cannot incorporate lipophilic drugs due to its significant lipophobicity [40]. Previous reports showed the incorporation of therapeutic moieties in the surfactant layer surrounding PFC core of a nanoemulsion droplet [41], [42]. Alternatively, coconut oil was used to solubilize lipophilic drug camptothecin in a PFC emulsion [43]. Similarly, we used Miglyol 810N to solubilize celecoxib and a NIRF dye. Miglyol 810N, a medium-chain triglyceride of GRAS (generally regarded as safe) category, is widely used in parenteral nutrition emulsion formulations [44].

The challenging task of stabilizing immiscible hydrocarbon oil (Miglyol 810N) and PFPE was achieved by using a combination of nonionic surfactants CrEL and P105 under high shear liquid processing (microfluidization). P105 belongs to Pluronic® block copolymers (of ethylene oxide and propylene oxide subunits) which are commonly used for solubilization of hydrophobic drugs [45]. CrEL, produced by reacting castor oil and ethylene oxide in 1∶35 molar [46] is used in pharmaceutical preparations as solubilizer for hydrophobic drugs and emulsifying agent [47], [48]. It is important to rationalize the use of CrEL in this formulation, because of the studies showing associated toxicity. CrEL is associated with hypersensitivity reactions, hyperlipidemia, abnormal lipoprotein patterns, aggregation of erythrocytes and peripheral neuropathy which were observed with paclitaxel formulation, Taxol [47]. The amount of CrEL in Taxol is as high as 26 mL per administration, with each mL of formulation containing 527 mg of CrEL [47], [49]. Paclitaxel formulations with reduced amount of CrEL showed significantly decreased allergic reactions suggesting that CrEL related toxicity is dose dependent [50], [51]. The formulation reported in this work used only 13.8 mg of CrEL per mL emulsion, which is significantly lower (∼38 fold compared to Taxol). Based on these calculations and prior reports [47], we suspect that allergic reactions are unlikely with the PFPE formulations reported here. Nonetheless, the detailed toxicity studies in animal models is warranted and is part of future studies. Each component in this design has a unique role in achieving theranostic potential of the final nanoemulsion. The formulation ingredients were chosen to achieve a stable formulation of immiscible PFPE and hydrocarbon oil with dual imaging capabilities and drug delivery.

### Nanoemulsion preparation and characterization

**Figure 2:**
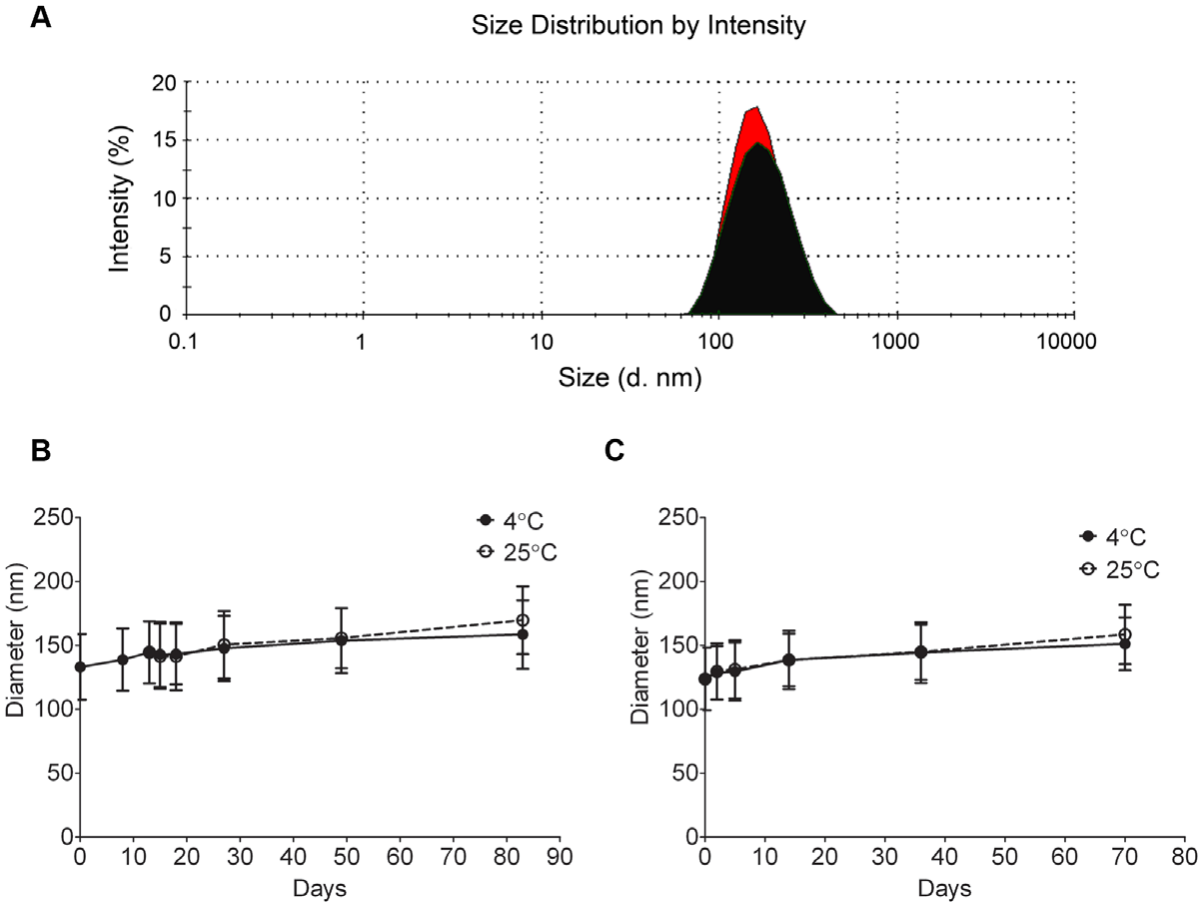
Shelf life of nanoemulsions with average droplet diameter (nm) at 4°C and 25°C. (A) Representative size distribution by intensity of nanoemulsions **A** (black) and **B** (red). (B) Stability of nanoemulsion **A**. (C) Stability of nanoemulsion **B**. Error bars represent half width of polydispersity index (PDIw/2).

Nanoemulsions with and without drug/dye (**B**, **C** and **A** respectively; Table 1) were prepared using high pressure liquid processing on microfluidizer M110S (Microfluidics Corp. Newton, MA). Nanoemulsion **A** acts as a drug and dye free control for nanoemulsion **B**; nanoemulsion **C** (containing Cellvue® Burgundy) was formulated to obtain confocal images of labeled cells due to the unavailability of confocal excitation laser for Cellvue® NIR815. During processing, use of organic solvents and thin film emulsification method was avoided as residual solvents in the final formulation could lead to cell toxicity in test cultures. DLS measurements showed an average droplet size and polydispersity index (PDI) of less than 160 nm and 0.15 respectively. Shelf life was determined by following the droplet size and PDI upon storage at 4°C and 25°C (Figure 2). The inclusion of drug and dye in the nanoemulsion had no significant effect (p = 0.1275, Mann Whitney test, GraphPad Prism) on droplet size over time upon storage at 4°C (Figure 2). Nanoemulsions **A** and **B** were stable for at least 70 days. However, when stored at 25°C minimal average size increase was observed. Therefore, the nanoemulsions are recommended to be stored at 4°C. A representative size distribution graph of nanoemulsions **A** and **B** is shown in Figure 2A. Small droplet size helps end-process sterilization by filtration [51] which is needed for future *in vivo* experiments. To further evaluate stability, zeta potential of the nanoemulsions was measured. Large values of zeta potential (> ±30 mV) ensure greater repulsion between the nanodroplets leading to a stable nanoemulsion [52]. Both drug free and drug loaded nanoemulsions, sterically stabilized by nonionic surfactants, showed a moderate zeta potential value around −17±6 mV (Figure S1).

Nanoemulsions were further characterized by ^19^F NMR and NIRF imaging. In nanoemulsions **A** and **B**, ^19^F NMR peak at −91.5 ppm has not shown any changes in chemical shift and line shape ([38]; Figures S2 and S3). This result demonstrates the chemical stability of PFPE in the final product and during processing. ^19^F NMR and NIRF images were recorded for nanoemulsion **B** dilutions in deionized water. NIRF images of nanoemulsion **B** are shown in Figure S4 and signal intensities in Table S1. A linear relationship was obtained for fluorine nuclei and NIRF signal for the dilution series (Figure S5). Based on this result, we believe that the estimates of *in vitro* cell labeling can be obtained by NIRF imaging alone without the need for ^19^F NMR.

Reverse phase HPLC was utilized to evaluate drug loading in nanoemulsion **B**. All the formulation ingredients were individually run for any possible interference with the celecoxib peak. Excipients did not show UV absorbance around 252 nm (data not shown). Predicted celecoxib concentration based on calibration model was found to be 139.3±8.7 µg/mL nanoemulsion. To summarize, DLS results confirm the formation of nanoemulsion with stable droplet size. ^19^F NMR and NIRF imaging clearly showed the incorporation of PFPE and NIRF dye in the nanoemulsion. HPLC analysis quantified the drug content in nanoemulsion **B**.

### *In vitro* toxicity and uptake studies in macrophages

**Figure 3:**
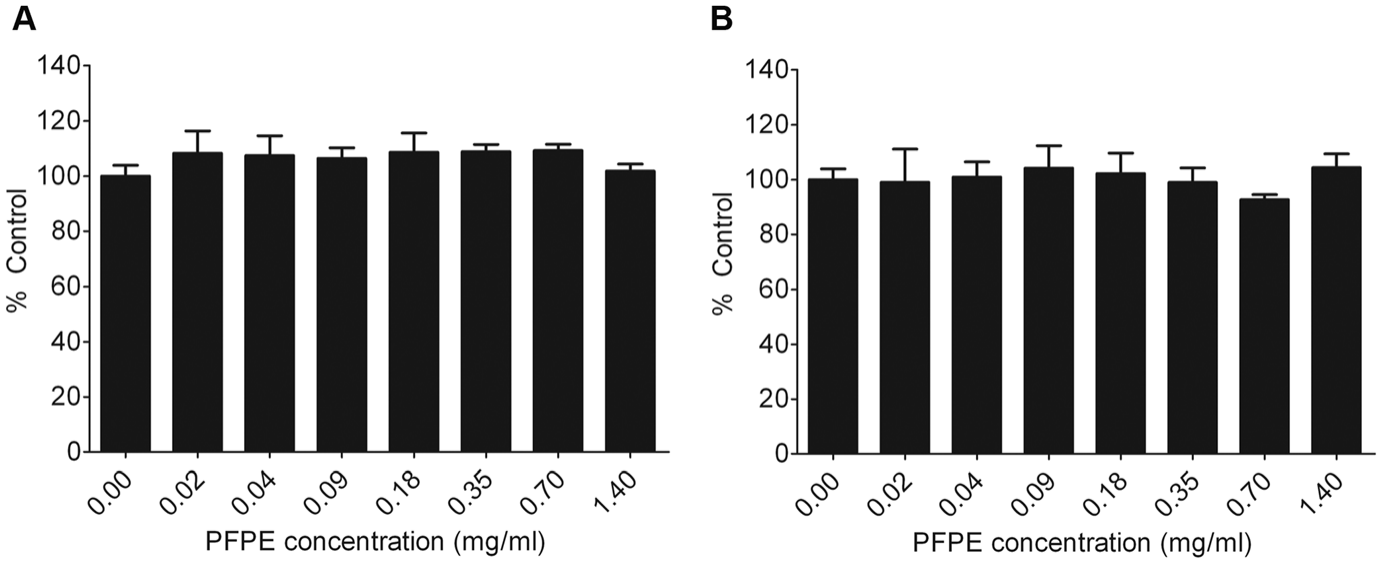
Macrophage cell viability post labeling. (A) Nanoemulsion **A** (B) Nanoemulsion **B**. Each data point represent mean of at least three replicates and the error bars are standard deviation of the mean. Values are reported as percent control (0 mg/mL PFPE).

Before performing *in vitro* biological tests, colloidal stability of nanoemulsions in cell culture medium was evaluated by monitoring changes in droplet size. Nanoemulsions **A** and **B** were incubated in the complete cell culture medium for 24 h. No considerable change in droplet size and PDI was noted under cell culture relevant conditions (Figure S6 and Table S2). This is a crucial finding as any structural changes in the nanodroplets during incubation with cells could give misleading results on the nanodroplet cellular uptake and toxicity profile, which would further render the nanoemulsions unsuitable for *in vivo* testing. With this result, *in vitro* toxicity studies were conducted using Celltiter-Glo® Luminescence Cell Viability Assay to assess the suitability of the prepared nanoemulsions for biomedical applications. The assay makes use of the amount of ATP present in the culture to quantitate the number of metabolically active or viable cells. Mouse macrophages (RAW 264.7) were chosen as the model inflammatory cells. As shown in Figure 3, no considerable effect on cell viability was detected after 24 h exposure to nanoemulsions. Cell viability was between 92–104% of the control group (untreated cells).

**Figure 4:**
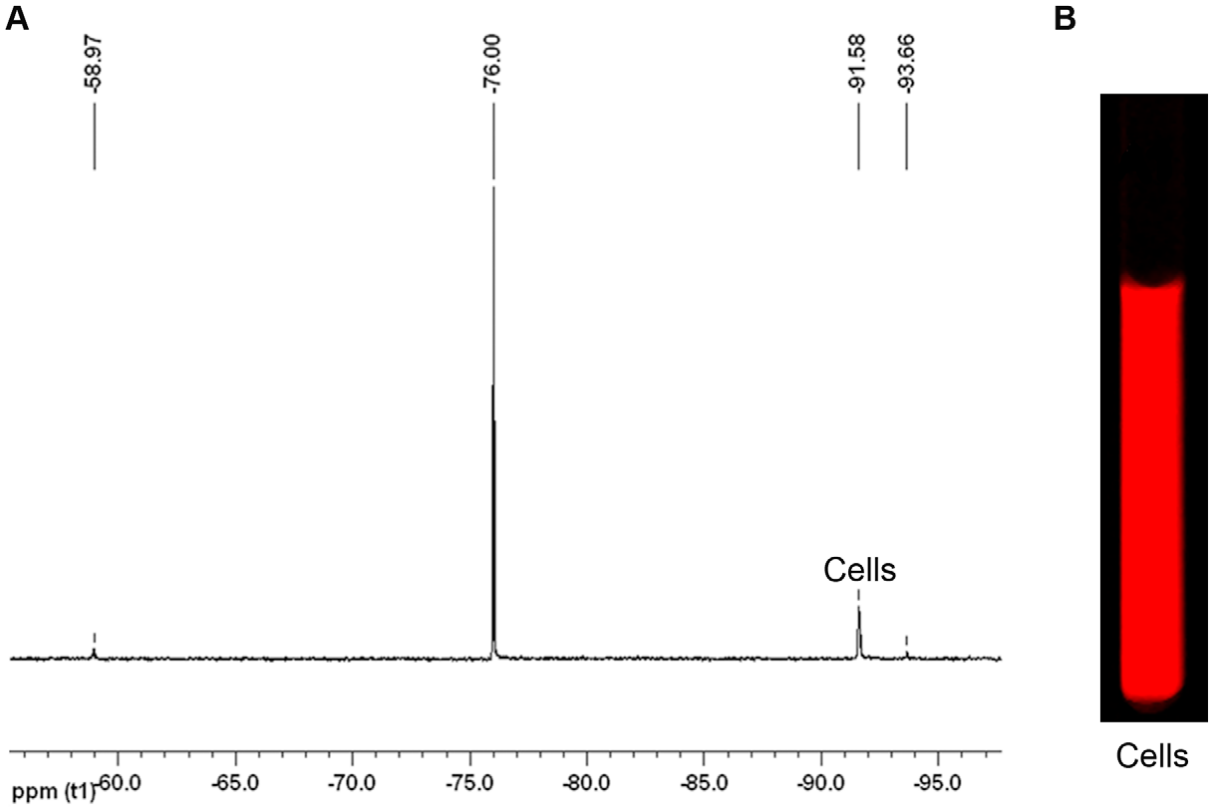
^19^F NMR and NIRF imaging of nanoemulsion B labeled macrophages. (A) ^19^F NMR of cells labeled with nanoemulsion **B**. 0.02% v/v aqueous TFA set at −76.00 ppm was used as reference for ^19^F NMR. (B) NIRF image (at 800 nm) of cells labeled with nanoemulsion **B** in NMR tube.

**Figure 5:**
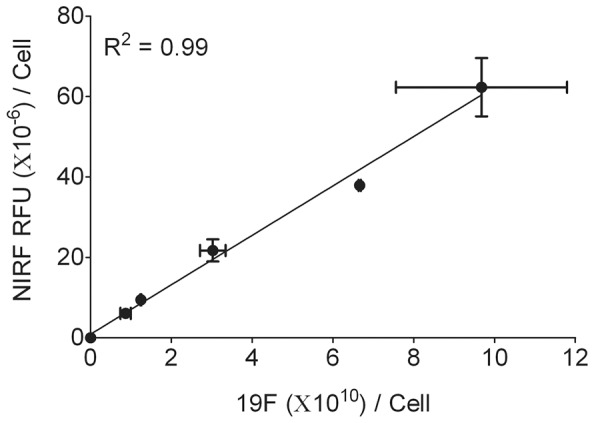
^19^F NMR-NIRF correlation of labeled macrophage cells. Data points represent cells labeled with different concentrations of nanoemulsion **B** (0–1.4 mg/mL PFPE).

To investigate the utility of nanoemulsion **B** for future *in vivo* imaging studies, *in vitro* cell uptake tests were performed on macrophages. Macrophages were incubated with nanoemulsion **B** at different PFPE concentrations for 24 h and exposed cells were analyzed by ^19^F NMR and NIRF imaging to assess the intracellular uptake. Representative ^19^F NMR and NIRF image of labeled cells is shown in Figure 4. As shown in Figure 4A, PFPE line shape and peak position at −91.5 ppm was unchanged upon uptake in cells when compared with PFPE in nanoemulsion **B** (Figure S3). This result suggests the chemical stability of PFPE in cells which is crucial for their use as an imaging tracer. ^19^F NMR and NIRF measurements of labeled cells showed a dose-dependent uptake of the nanoemulsion (Figure S8). NIRF signal intensities and images of nanoemulsion **B** labeled cells at different dilutions is shown in Table S3 and Figure S7 respectively. Macrophages labeled with varying concentrations of nanoemulsion **B** showed a linear correlation (R^2^ = 0.99) between ^19^F signal and NIRF intensity per cell (Figure 5). Interestingly, linear correlation was obtained without chemically conjugating PFPE and fluorescent dye as reported earlier [29]. Based on these results, it can be proposed that the nanoemulsion was not destabilized before entering the cell in the labeling medium. Any instability of nanoemulsion would lead to poor or no correlation between ^19^F NMR and NIRF signals due to the differences in uptake of imaging agents. A strong correlation between signals corresponding to two imaging agents is a requisite to utilize the nanoemulsion for *in vitro* and *in vivo* dual mode imaging studies.

*In vitro* ^19^F NMR was used to evaluate the utility of the nanoemulsion for future *in vivo* ^19^F MRI. Presented nanoemulsions have lower amount (7.2% w/v) of PFPE than our earlier reported cell tracking formulations [37], [53]. However, we found that at a very low PFPE concentration of only 1.4 mg/mL, significant cell uptake (1.0×10^11^ fluorine atoms per cell) is achieved. With this labeling efficiency, approximately 7.5×10^5^ cells per voxel are required to obtain *in vivo* ^19^F MR images at 11.7 T [27], [36]. These findings strongly suggest that we would be able to detect our nanoemulsion accumulation *in vivo*. Detailed dosing studies of the reported celecoxib loaded PFPE nanoemulsion in preclinical animal models are beyond the scope of this report and will be reported in the future.

**Figure 6:**
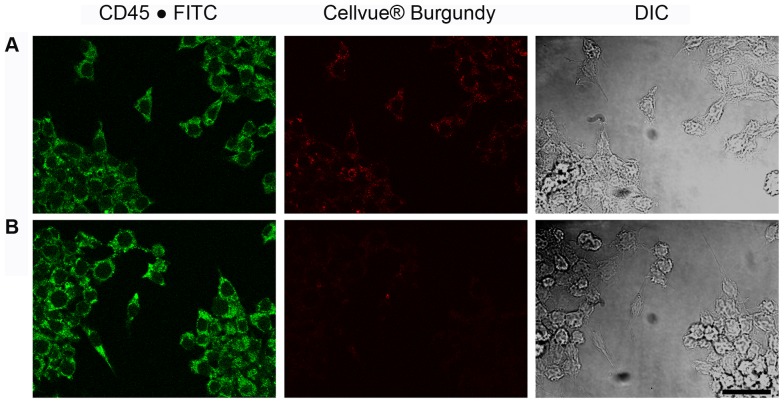
Fluorescence images of macrophages. (A) Cells labeled with anti-CD45 (FITC) green and incorporated nanoemulsion **C** containing celecoxib and Cellvue® Burgundy dye represented as red. (B) Cells not exposed to the nanoemulsion **C** exhibit CD45 labeling with FITC (green) but no red signal. Transmitted light DIC image acquired simultaneously shows field of view (Bar = 30 µm). The microscope image acquisition parameters were identical between the experimental and control, and the images were all acquired within 15 min of one another.

**Figure 7:**
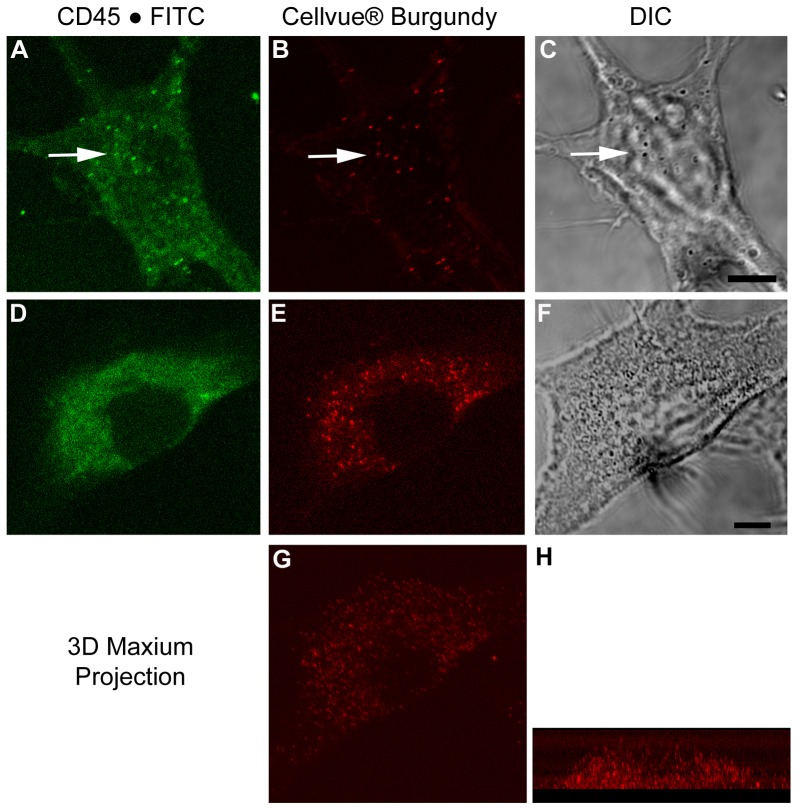
Magnified fluorescence images of individual macrophages exposed to nanoemulsion C. (A) Cells labeled with CD45-FITC (green) and incorporated nanoemulsion **C** containing celecoxib and Cellvue® Burgundy dye exhibit broad expression of CD45 as well as localized points of fluorescent signal indicating internalization of CD45 protein. (B) The same cell and focal plane as viewed in panel A reveals the internalized Cellvue® Burgundy labeled nanoemulsions as discrete particles. (C) The transmitted light DIC view of the cell reveals the black refractive droplets, coincident with the red and green fluorescent signals (Bar = 5 µm). (D) A different cell labeled with CD45-FITC (green), (E) internalized Cellvue® Burgundy (red) and (F) transmitted light DIC view reveals discrete droplets (Bar = 5 µm). (G) The cell shown in panel D was imaged in serial section and rendered by maximum-projection to represent all of the Cellvue® Burgundy labeled particles viewed from above and (H) in 90° cross-section, to reveal that the droplets are distributed throughout the cell cytoplasm.

Although ^19^F NMR and NIRF imaging of the nanoemulsion labeled cells showed dose-dependent cell labeling, conclusions about membrane adsorbed versus phagocytosed nanodroplets cannot be made from this data alone. Therefore, fluorescence confocal microscopy was performed on macrophages exposed to nanoemulsion. To more closely match the excitation and emission capabilities of the confocal microscope system, an alternate drug loaded nanoemulsion (nanoemulsion **C**, Table 1) was prepared with Cellvue® Burgundy dye (683 nm/707 nm). *In vitro* characterization of nanoemulsion **C** is shown in Figure S9. Cells exposed to nanoemulsion **C** were stained with anti-CD45.1 antibody conjugated with FITC dye (CD45-FITC). CD45 is a protein tyrosine phosphatase, receptor type C cell membrane associated protein. The nanoemulsion uptake was visualized by Cellvue® Burgundy dye. Figure 6A clearly shows the presence of the CD45 protein (green) and the Cellvue® Burgundy labeled nanodroplets (red) in the cytoplasm. As a control, cells not exposed to nanoemulsion were labeled with CD45-FITC (Figure 6B). No evidence of NIRF signal was observed in the control group. Figure 7 shows a higher magnification view of individual cells that reveal the particle nature of the Cellvue® Burgundy labeled nanodroplets, which are also evident in the transmitted DIC view of the cells as black refractive particles (Figure 7C, F). Three-dimensional rendering of a z series of 33 optical sections for the 10.4 µm thickness of the cell revealed that the nanodroplets are within the cytoplasm, specifically in the maximum projection cross-sectional view of the cell (Figure 7H). It is interesting to note that the endocytic engulfment of the nanodroplet has also internalized the CD45 protein and many of the nanodroplets co-present with green and red fluorescent signals. CD45 internalization has been previously reported [54]. ^19^F NMR, NIRF imaging and confocal fluorescence microscopy clearly demonstrates the uptake of nanoemulsion droplets by exposed macrophages *in vitro*. In a separate experiment, presence of nanoemulsion droplets in the intracellular compartments was assessed by labeling lysosomes of macrophages with Lysotracker® Green (Protocol S1). It appears that nanoemulsion droplets are distributed in the entire volume of the cytoplasm and no preferential accumulation in the lysosomes was observed (Figure S11).

### COX-2 inhibition in macrophages

**Figure 8:**
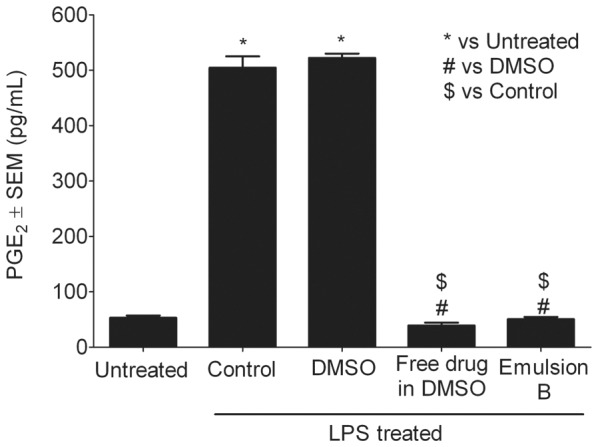
Production of PGE_2_ in macrophages assessed after LPS treatment. LPS treatment was performed post cell labeling with nanoemulsion **B**, free drug dissolved in DMSO and DMSO. Cells not exposed to LPS were designated as untreated. * # $ represents statistical significance comparisons (p<0.0001) between treatments. Each data point represents the average of four independent measurements, where the error bars are the standard error of the mean (SEM).

Macrophages in the tumor environment express elevated levels of COX-2 which is involved in the biosynthesis of PGE_2_. The potential anti-inflammatory effect of celecoxib loaded nanoemulsion **B** on the production of PGE_2_ by LPS activated macrophages was studied. Macrophages were first exposed to nanoemulsion **B** for 24 h, washed with medium and then activated using LPS for 4 h. Amount of PGE_2_ released into the medium was quantified using a commercially available ELISA assay. One-way ANOVA with Tukey's multiple comparison test was employed to evaluate the statistical significance between the treatments. Results are shown in Figure 8. LPS activated cells showed up to 10 fold increase in PGE_2_ as compared to untreated and a statistically significant difference was observed (p<0.0001). A statistically significant difference (p<0.0001) between nanoemulsion **B** and LPS treated control was observed. Cells labeled with nanoemulsion **B** produced, on average, 50.6±8.2 pg of PGE_2_ per mL as compared to 504.5±41.2 pg/mL by LPS activated control. Exposing LPS activated macrophages to DMSO (vehicle for free drug) has not shown any effect on PGE_2_ production compared to control. Although, PGE_2_ reduction by nanoemulsion **B** is not statistically different from free drug, nanoemulsion mediated celecoxib delivery may be advantageous in reducing systemic exposure to the drug and related side effects. Additionally, dual mode imaging capabilities allow for non-invasive imaging of nanoemulsion biodistribution. In a separate experiment, effect of nanoemulsions **A** and **B** on PGE_2_ production was studied. Nanoemulsion **B** showed significant reduction in PGE_2_ production compared to nanoemulsion **A** and control (Figure S10). Nanoemulsion **A** has not shown any significant contribution to changes in PGE_2_ levels proving that the drug free vehicle is inert towards PGE_2_ production. The presented theranostic PFPE nanoemulsion showed celecoxib delivery and COX-2 inhibition in macrophages. Introduction of celecoxib directly to macrophages by nanoemulsions loading may change their phenotype from tumor promoting M2 to tumor suppressing M1-like.

## Conclusion

This paper presented novel drug carrying nanoemulsion formulation equipped with dual mode (^19^F MR and NIRF) imaging capabilities. The prepared nanoemulsions showed good stability for at least 70 days. The utility of dual mode imaging was shown by a strong correlation between NIRF and ^19^F NMR signals of labeled cells. Confocal imaging clearly demonstrated that the nanoemulsion droplets are incorporated into the cytoplasm of engulfing cells. Nanoemulsion delivery of celecoxib is demonstrated in macrophages by their inhibitory effect on PGE_2_ production and release. The formulation platform developed here can be used to incorporate other lipophilic drugs and can act as a dual imaging tracer to label phagocytic cells such as macrophages. Drug release and *in vitro/in vivo* activity studies on breast cancer models are currently under investigation.

## Supporting Information

Equation S1 **PFPE amount (mg/mL nanoemulsion) calculation.**
(DOC)

Figure S1 **Zeta potential distribution as measured by Zetasizer Nano (Malvern, UK).** Zeta potential of nanoemulsion **A** (red, −17±6.6 mV) and nanoemulsion **B** (black, −17.7±6.7 mV) in deionized water at 1∶39 v/v dilution.
(TIF)

Figure S2 **Representative ^19^F NMR of nanoemulsion A.** NMR was recorded on Bruker Instruments, Inc., Billerica, MA at 470 MHz in water with TFA reference at −76.00 ppm.
(TIF)

Figure S3 **Representative ^19^F NMR of nanoemulsion B.** NMR was recorded on Bruker Instruments, Inc., Billerica, MA at 470 MHz in water with TFA reference at −76.00 ppm.
(TIF)

Figure S4 **Representative NIRF imaging of nanoemulsion B dilutions.** Decreasing concentration of the emulsion from left to right. Images at 785 nm excitation wavelength and emission above 810 nm were collected on Li-COR Odyssey® Infrared imaging system in 5 mm Borosilicate NMR tubes. For NIRF signal intensity, see Table S1.
(TIF)

Figure S5 **Nanoemulsion B dilutions (1∶1 v/v) in 0.02% v/v TFA** (A) Plot of ^19^F atoms (of PFPE around −91.5 ppm) with percent emulsion in NMR sample. (B) Plot of NIRF RFU with percent emulsion in the NMR sample (5 mm borosilicate NMR tubes, 0.4 mL total volume).
(TIF)

Figure S6 **Stability of nanoemulsions in cell culture medium.** Droplet size distribution before incubation is shown in red and after incubation in black. (A) Nanoemulsion **A** (B) Nanoemulsion **B**. No significant change in dropletsize was seen for both nanoemulsions after 24 h incubation with cell culture medium (Table S2). Analysis performed on Zetasizer Nano (Malvern, UK).
(TIF)

Figure S7 **Representative NIRF imaging of cells labeled with nanoemulsion B.** Images at 785 nm excitation wavelength and emission above 810 nm were collected on Li-COR Odyssey® Infrared Imaging system in 5 mm Borosilicate NMR tubes. For NIRF signal intensity, see Table S3.
(TIF)

Figure S8 **Dose dependent uptake of nanoemulsion B.** (A) ^19^F atoms/cell at different concentrations of PFPE. (B) NIR fluorescence/cell at different concentrations of PFPE.
(TIF)

Figure S9 **Characterization of nanoemulsion C.** Nanoemulsion **C** was prepared to facilitate confocal microscopy. (A) Stability at 4°C and 25°C (B) Macrophage viability post 24 h exposure.
(TIF)

Figure S10 **Production of PGE_2_ in activated macrophages.** Macrophages were exposed to either of the nanoemulsions **A** and **B** at 1.4 mg/mL PFPE concentration. LPS treatment was performed post cell labeling with nanoemulsions **A** or **B** for 3 h. Fresh medium was added to unexposed cells (untreated). Control represents LPS activated unexposed cells. PGE_2_ production was quantified in the supernatant using PGE_2_ ELISA kit (Cayman Chemicals). Each data point represents the average of at least nine independent measurements, where the error bars are the standard error of the mean (SEM). Statistically significant difference was obtained between nanoemulsion **B** and all other treatments. One-way ANOVA with Tukey's multiple comparison test was conducted to evaluate statistical significance.
(TIF)

Figure S11 **Fluorescence microscopy of macrophages exposed to nanoemulsion C and lysosome specific fluorescent probe.** (A) The transmitted light DIC view of the cells; (B) Fluorescent image of nucleus (blue) and lysosomes (green); (C) Fluorescent image of nucleus (blue) and nanoemulsion **C** (red) and (D) Fluorescent image of nucleus, lysosomes and nanoemulsion droplets. The scale bar represents 10 µm.
(TIF)

Table S1 **NIR signal intensity (relative fluorescence units, RFU) of nanoemulsion B dilutions in 0.02% aq.** TFA (1∶1 v/v). Sample G represents nanoemulsion **A** (without NIRF dye) in aqueous TFA to correct for background.
(DOC)

Table S2 **Average droplet diameter and PDI of nanoemulsions A and B before and after incubation in media.**
(DOC)

Table S3 **NIR signal intensity (relative fluorescence units, RFU) of cells labeled with nanoemulsion B (A–E) and unlabeled control cells (F).** Unlabeled cells were used to correct for the background fluorescence signal from the cell suspension.
(DOC)

Protocol S1 **Lysosomal labeling and confocal imaging procedure.**
(DOC)
