# Supplementary material for: Cyclooxgenase-2 Inhibiting Perfluoropoly (Ethylene Glycol) Ether Theranostic Nanoemulsions—In Vitro Study
Source: PLoS One. 2013 Feb 7;8(2):e55802. doi: 10.1371/journal.pone.0055802 (PMC3567136; doi:10.1371/journal.pone.0055802)
Supplement: Equation S1 — PFPE amount (mg/mL nanoemulsion) calculation. (DOC) [file pone.0055802.s001.doc]

**Equation S1.** PFPE amount (mg/mL nanoemulsion) calculation

$\text{Amount of PFPE per mL}=\frac{5\left(\frac{Fne}{Fr}\right)Nr}{N*40}*Mwt$

*Fne* – Integrated PFPE peak value around -91.5 ppm ; *Fr* – Integrated reference peak value under -76 ppm; *Nr* – number of fluorine atoms in TFA used for analysis; *N -* Avogadro number (6.023x10^23^); Mwt – molecular weight of PFC (1380 for PFPE). 40 is the number of fluorine atoms around -91.5 ppm peak in PFPE ^19^F NMR. 5 is the multiplication factor (200 µL of nanoemulsion was used in NMR tube) to obtain the amount per mL nanoemulsion. (The formula is adapted from earlier ^19^F content calculations reported by Ahrens *et al*, Nature Biotechnology, 2005; Srinivas *et al*, Mag. Res. Med. 2007 and Janjic *et al*, J. Am. Chem. Society, 2008).
