# Supplementary figures and images for: Cyclooxgenase-2 Inhibiting Perfluoropoly (Ethylene Glycol) Ether Theranostic Nanoemulsions—In Vitro Study
Source: PLoS One. 2013 Feb 7;8(2):e55802. doi: 10.1371/journal.pone.0055802 (PMC3567136; doi:10.1371/journal.pone.0055802)

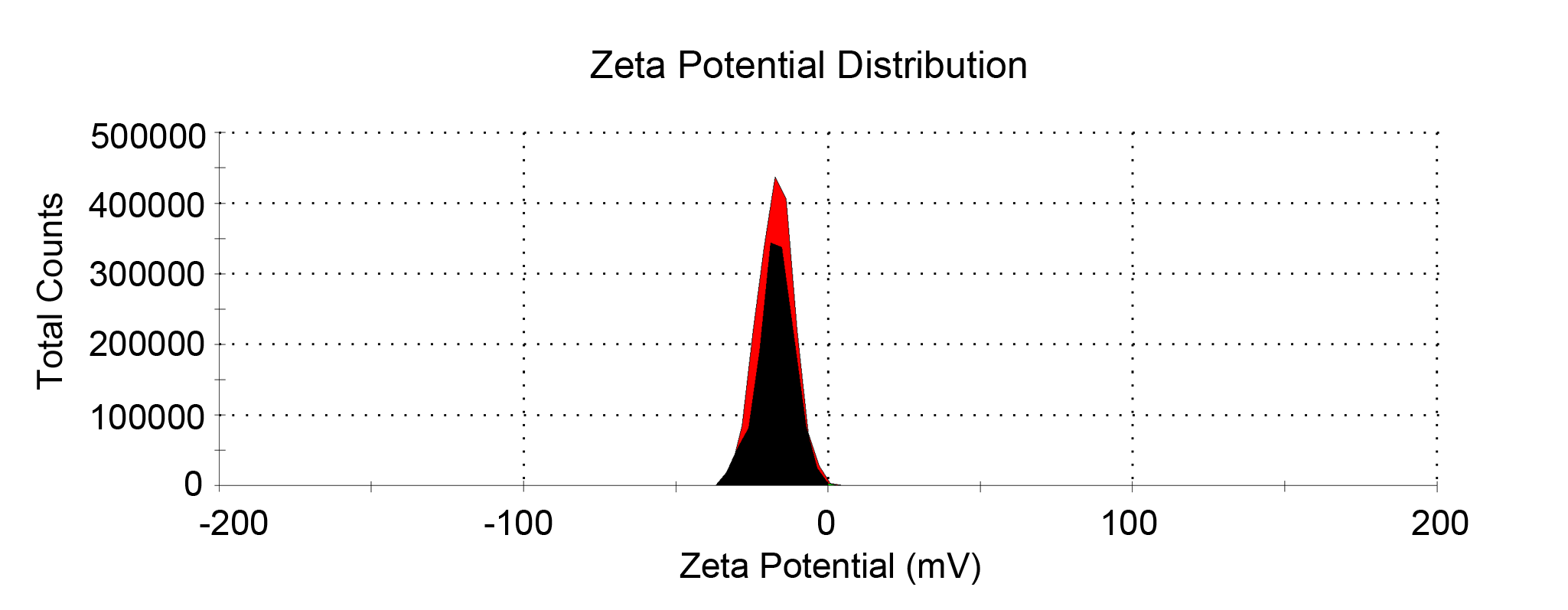

Supplement: Figure S1 — Zeta potential distribution as measured by Zetasizer Nano (Malvern, UK). Zeta potential of nanoemulsion A (red, −17±6.6 mV) and nanoemulsion B (black, −17.7±6.7 mV) in deionized water at 1∶39 v/v dilution. (TIF) [file pone.0055802.s002.tif]

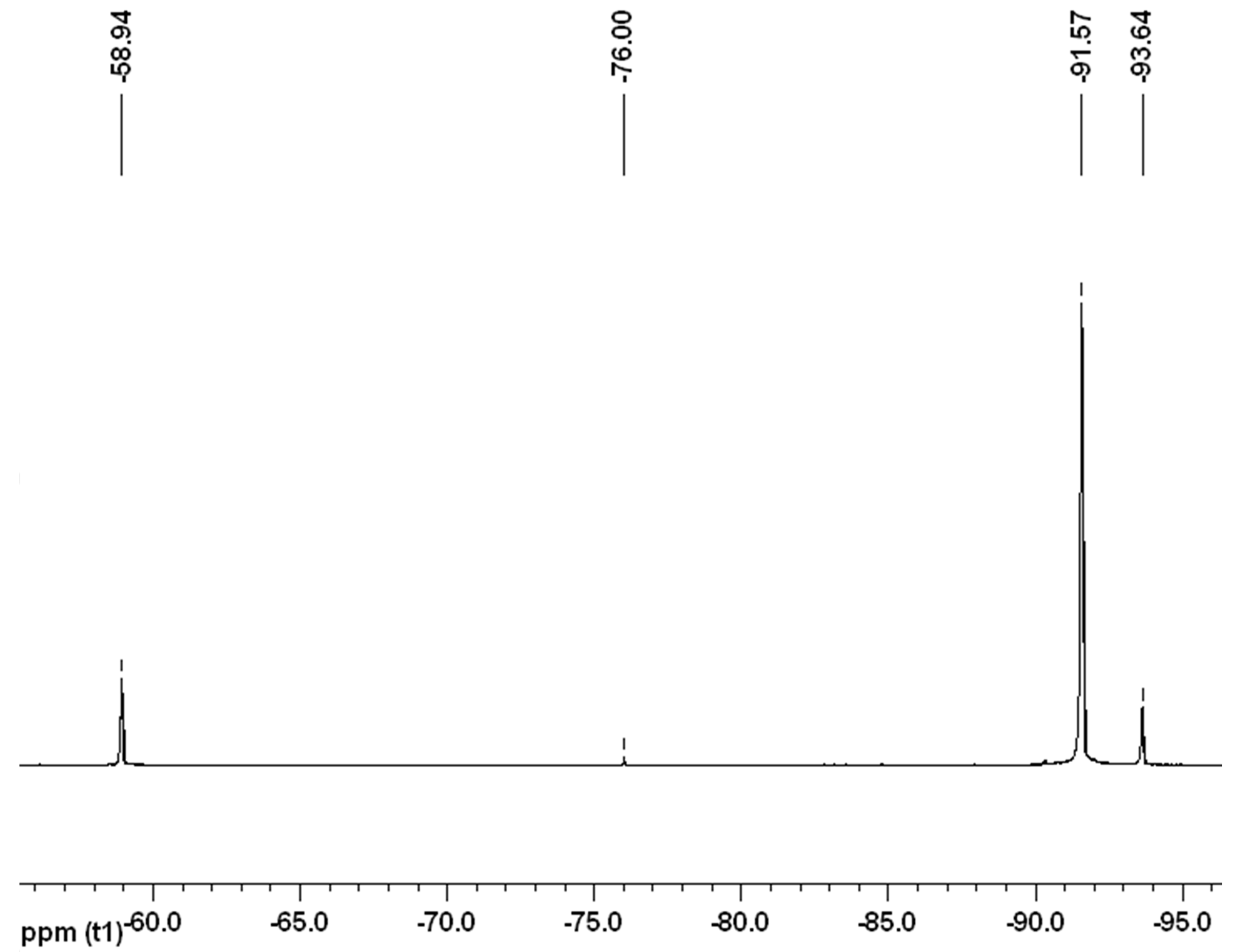

Supplement: Figure S2 — Representative 19F NMR of nanoemulsion A. NMR was recorded on Bruker Instruments, Inc., Billerica, MA at 470 MHz in water with TFA reference at −76.00 ppm. (TIF) [file pone.0055802.s003.tif]

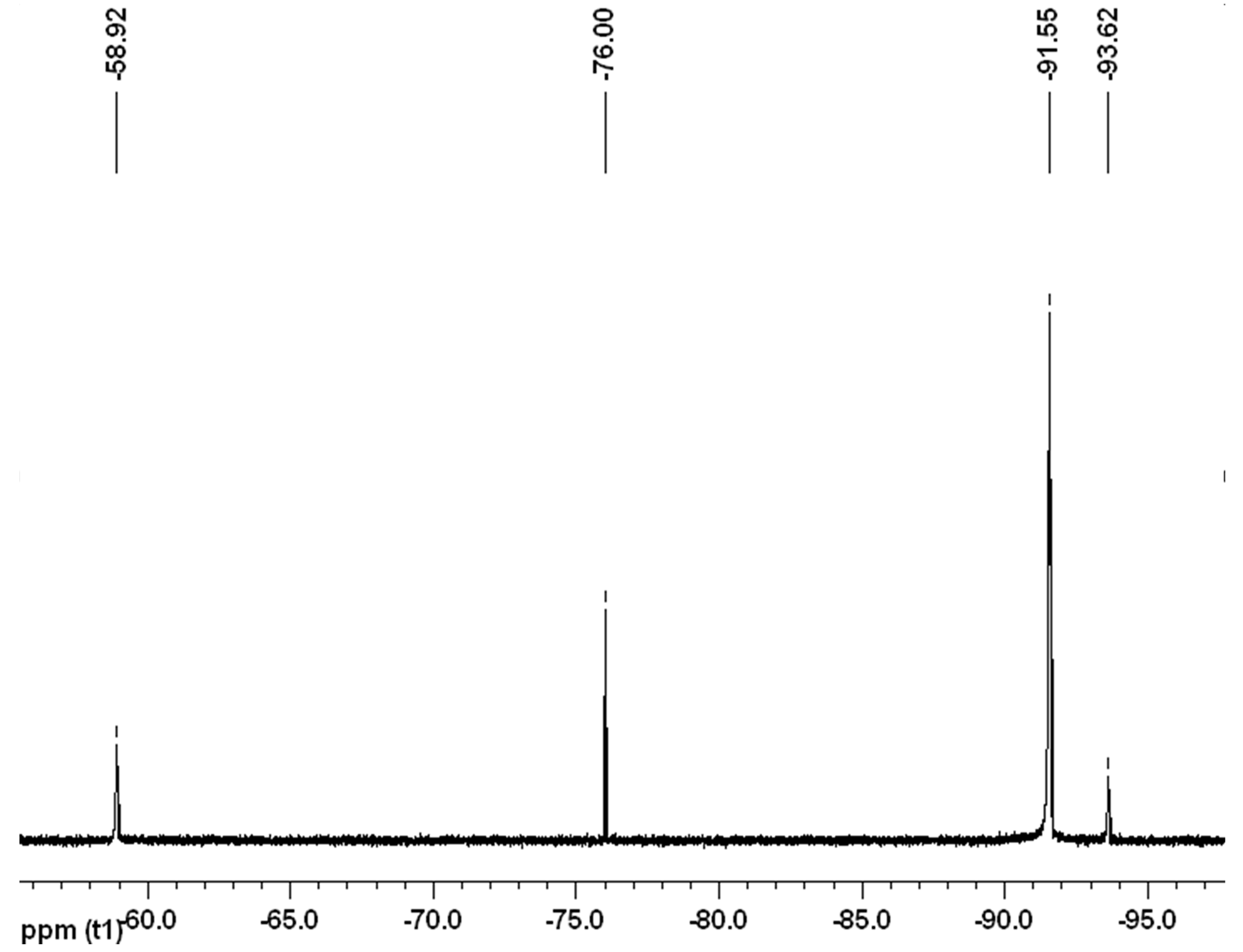

Supplement: Figure S3 — Representative 19F NMR of nanoemulsion B. NMR was recorded on Bruker Instruments, Inc., Billerica, MA at 470 MHz in water with TFA reference at −76.00 ppm. (TIF) [file pone.0055802.s004.tif]

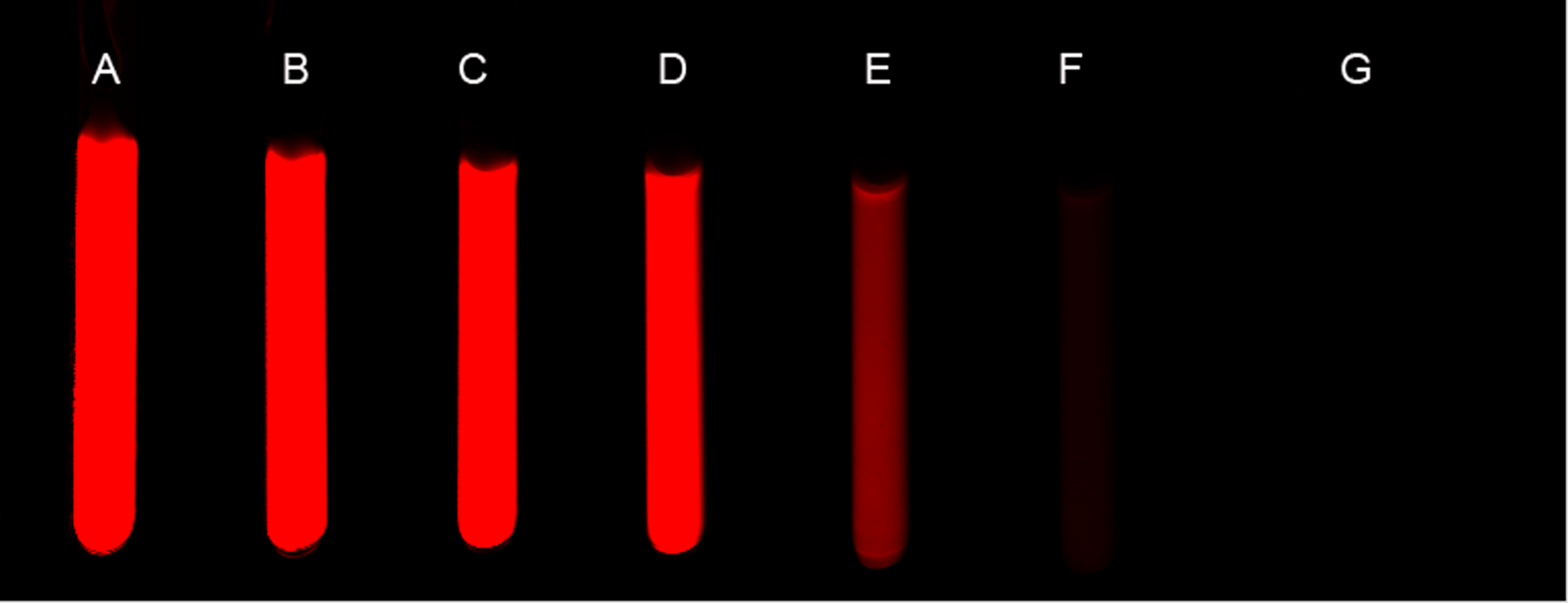

Supplement: Figure S4 — Representative NIRF imaging of nanoemulsion B dilutions. Decreasing concentration of the emulsion from left to right. Images at 785 nm excitation wavelength and emission above 810 nm were collected on Li-COR Odyssey® Infrared imaging system in 5 mm Borosilicate NMR tubes. For NIRF signal intensity, see Table S1. (TIF) [file pone.0055802.s005.tif]

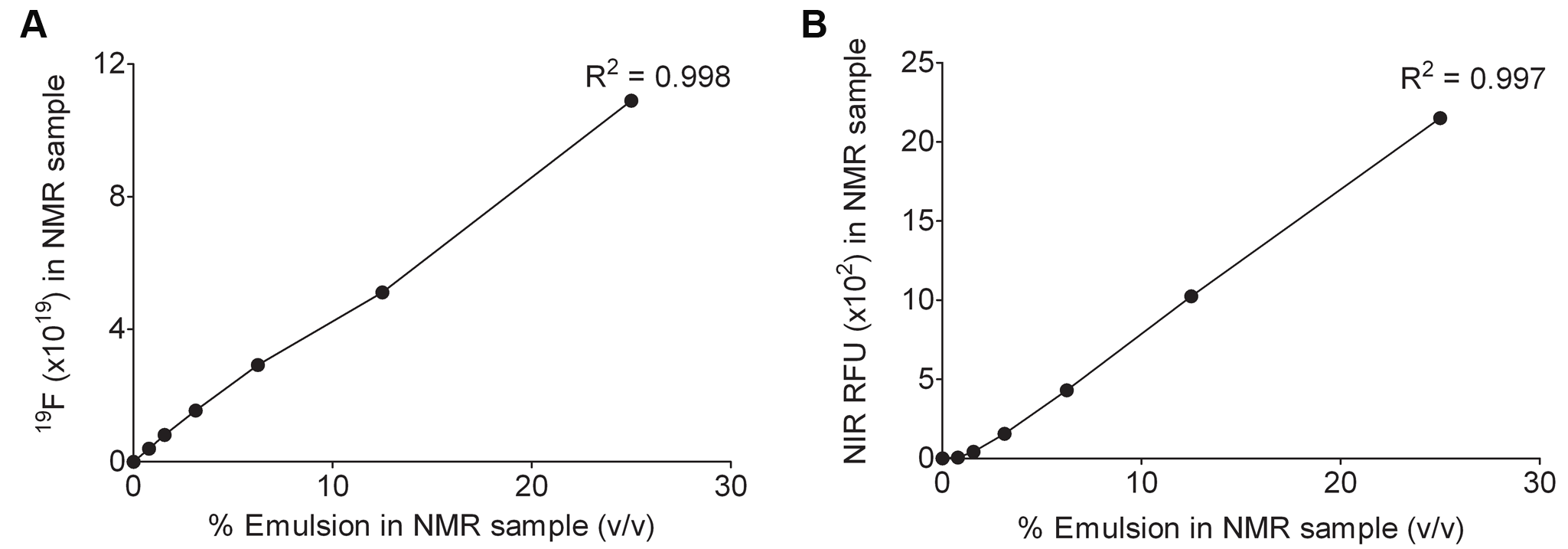

Supplement: Figure S5 — Nanoemulsion B dilutions (1∶1 v/v) in 0.02% v/v TFA (A) Plot of 19F atoms (of PFPE around −91.5 ppm) with percent emulsion in NMR sample. (B) Plot of NIRF RFU with percent emulsion in the NMR sample (5 mm borosilicate NMR tubes, 0.4 mL total volume). (TIF) [file pone.0055802.s006.tif]

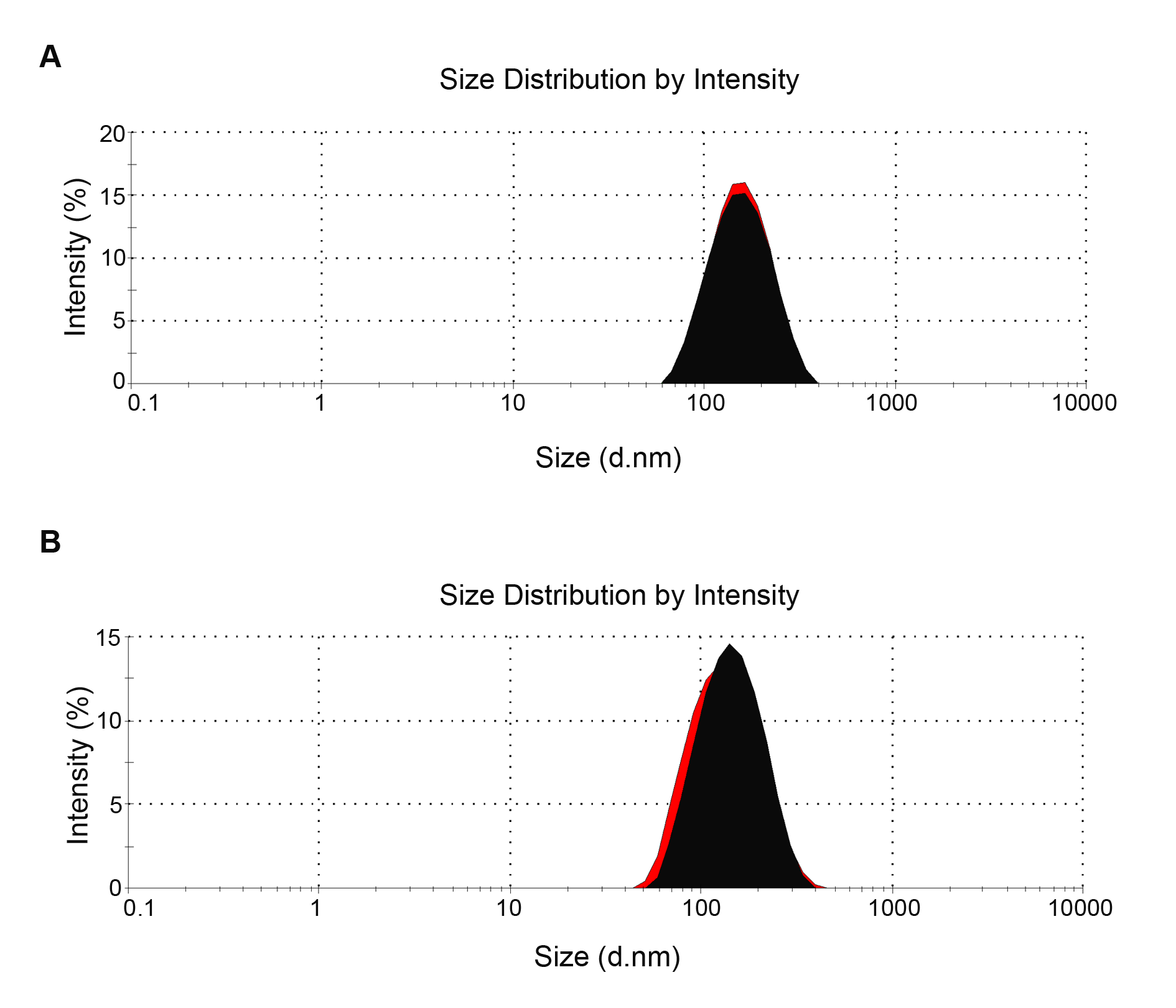

Supplement: Figure S6 — Stability of nanoemulsions in cell culture medium. Droplet size distribution before incubation is shown in red and after incubation in black. (A) Nanoemulsion A (B) Nanoemulsion B. No significant change in dropletsize was seen for both nanoemulsions after 24 h incubation with cell culture medium (Table S2). Analysis performed on Zetasizer Nano (Malvern, UK). (TIF) [file pone.0055802.s007.tif]

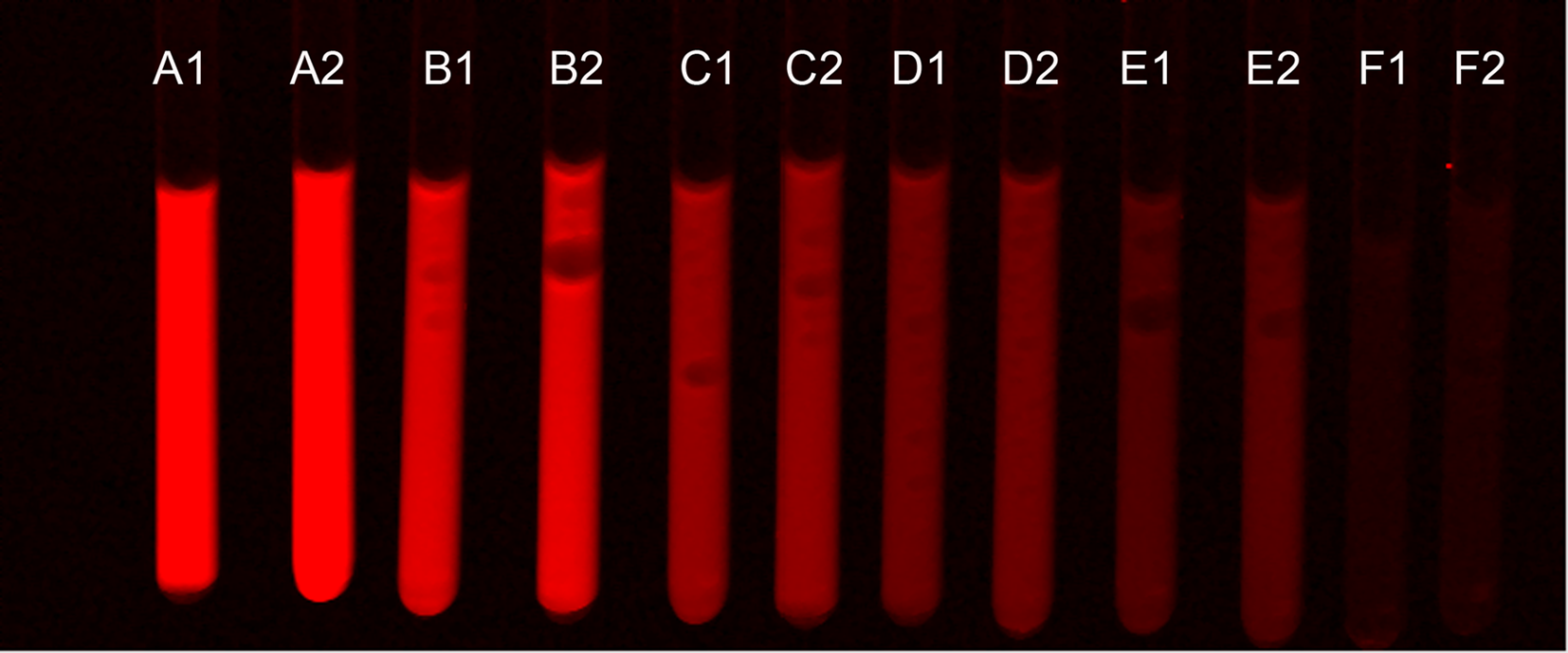

Supplement: Figure S7 — Representative NIRF imaging of cells labeled with nanoemulsion B. Images at 785 nm excitation wavelength and emission above 810 nm were collected on Li-COR Odyssey® Infrared Imaging system in 5 mm Borosilicate NMR tubes. For NIRF signal intensity, see Table S3. (TIF) [file pone.0055802.s008.tif]

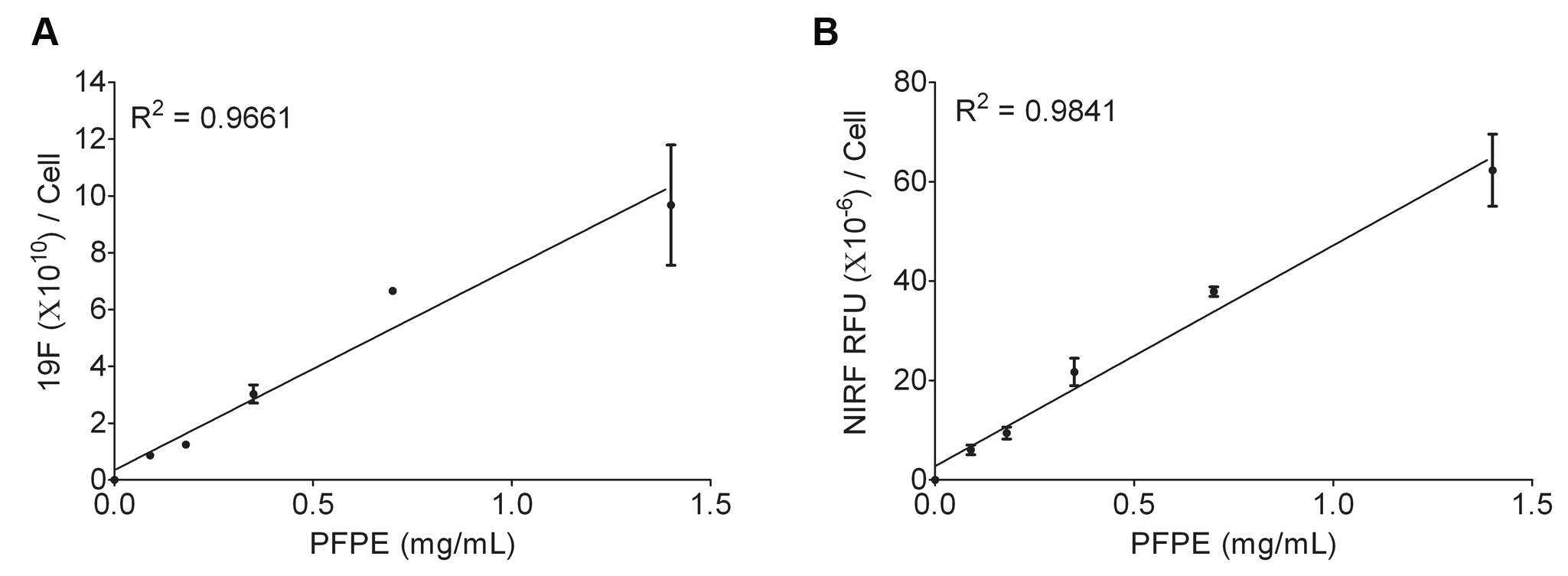

Supplement: Figure S8 — Dose dependent uptake of nanoemulsion B. (A) 19F atoms/cell at different concentrations of PFPE. (B) NIR fluorescence/cell at different concentrations of PFPE. (TIF) [file pone.0055802.s009.tif]

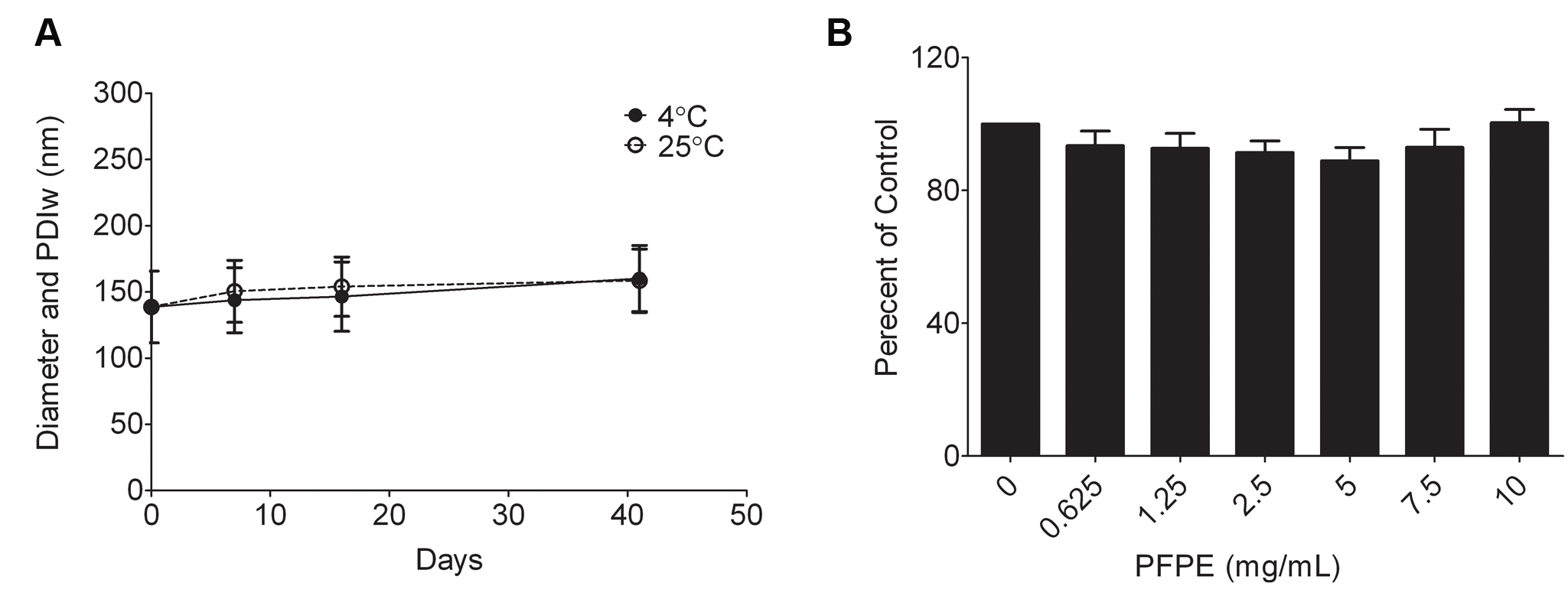

Supplement: Figure S9 — Characterization of nanoemulsion C. Nanoemulsion C was prepared to facilitate confocal microscopy. (A) Stability at 4°C and 25°C (B) Macrophage viability post 24 h exposure. (TIF) [file pone.0055802.s010.tif]

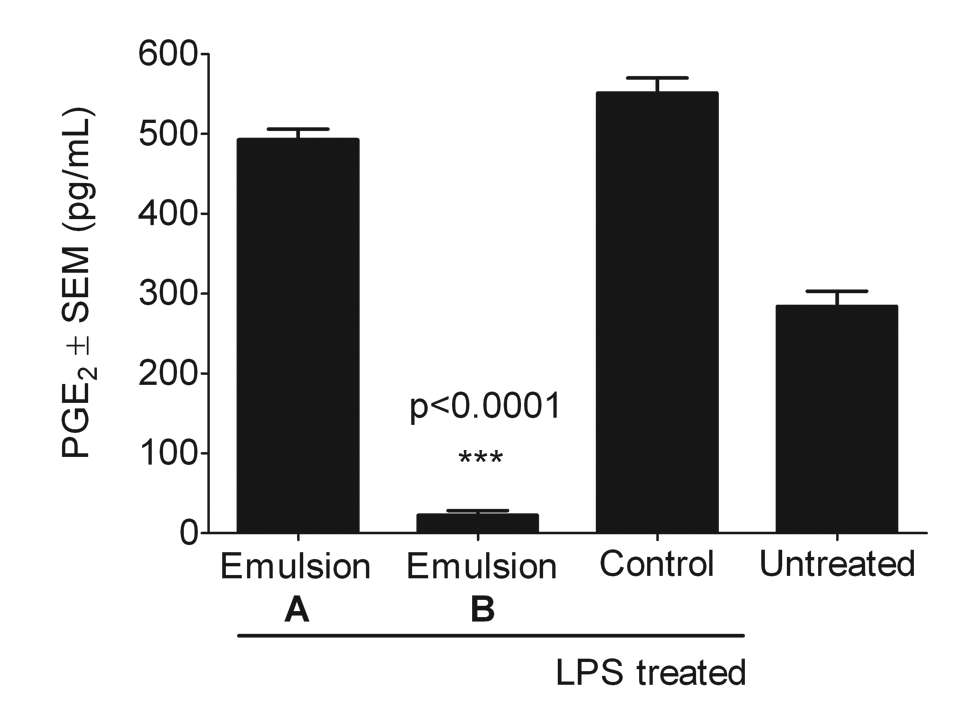

Supplement: Figure S10 — Production of PGE2 in activated macrophages. Macrophages were exposed to either of the nanoemulsions A and B at 1.4 mg/mL PFPE concentration. LPS treatment was performed post cell labeling with nanoemulsions A or B for 3 h. Fresh medium was added to unexposed cells (untreated). Control represents LPS activated unexposed cells. PGE2 production was quantified in the supernatant using PGE2 ELISA kit (Cayman Chemicals). Each data point represents the average of at least nine independent measurements, where the error bars are the standard error of the mean (SEM). Statistically significant difference was obtained between nanoemulsion B and all other treatments. One-way ANOVA with Tukey's multiple comparison test was conducted to evaluate statistical significance. (TIF) [file pone.0055802.s011.tif]

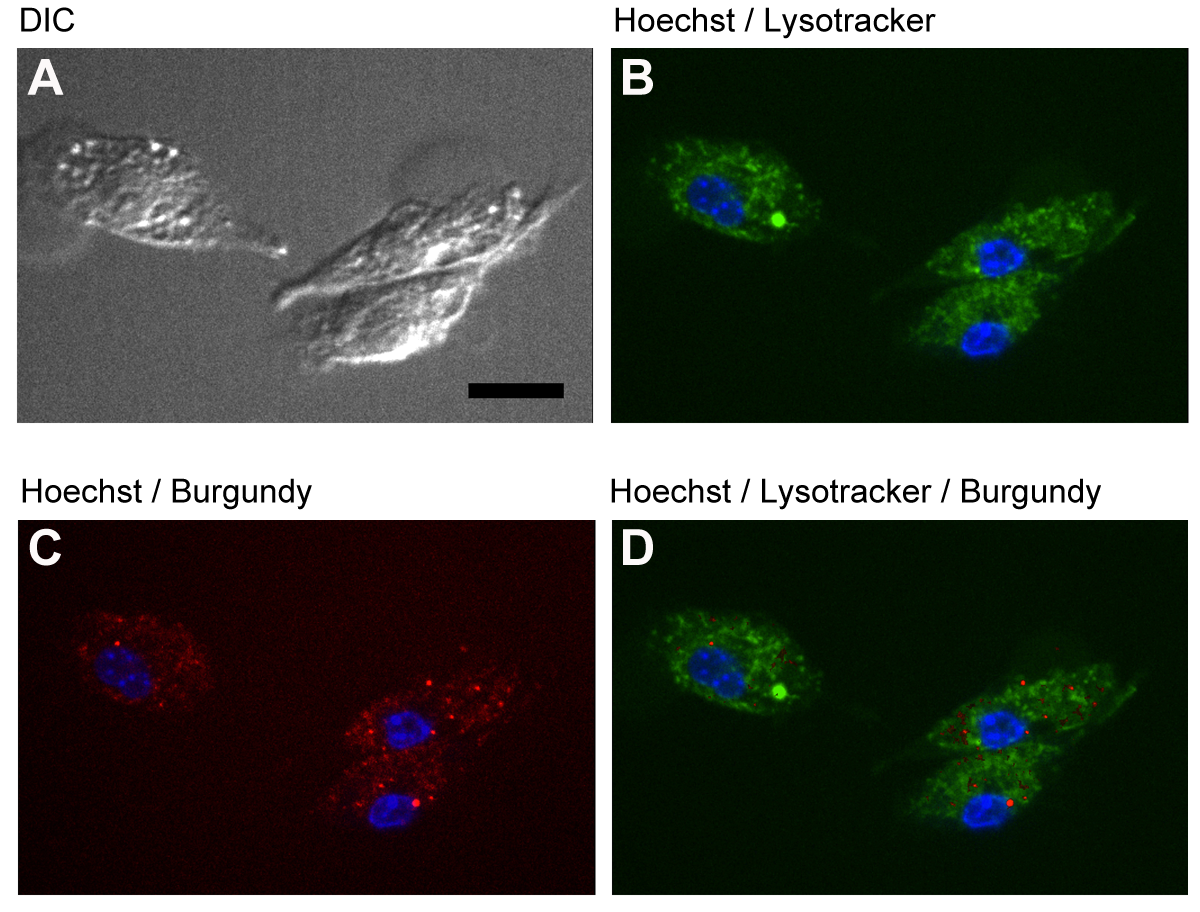

Supplement: Figure S11 — Fluorescence microscopy of macrophages exposed to nanoemulsion C and lysosome specific fluorescent probe. (A) The transmitted light DIC view of the cells; (B) Fluorescent image of nucleus (blue) and lysosomes (green); (C) Fluorescent image of nucleus (blue) and nanoemulsion C (red) and (D) Fluorescent image of nucleus, lysosomes and nanoemulsion droplets. The scale bar represents 10 µm. (TIF) [file pone.0055802.s012.tif]
