# Supplementary material for: Cyclooxgenase-2 Inhibiting Perfluoropoly (Ethylene Glycol) Ether Theranostic Nanoemulsions—In Vitro Study
Source: PLoS One. 2013 Feb 7;8(2):e55802. doi: 10.1371/journal.pone.0055802 (PMC3567136; doi:10.1371/journal.pone.0055802)
Supplement: Table S1 — NIR signal intensity (relative fluorescence units, RFU) of nanoemulsion B dilutions in 0.02% aq. TFA (1∶1 v/v). Sample G represents nanoemulsion A (without NIRF dye) in aqueous TFA to correct for background. (DOC) [file pone.0055802.s013.doc]

**Table S1**

NIR signal intensity (relative fluorescence units, RFU) of nanoemulsion **B** dilutions in 0.02% aq. TFA (1:1 v/v). Sample G represents nanoemulsion **A** (without NIRF dye) in aqueous TFA to correct for background.

| Sample | % Emulsion in total sample volume (with aq TFA) | NIR RFU |
| --- | --- | --- |
| A | 25 | 2150.82 |
| B | 12.5 | 1025.21 |
| C | 6.25 | 430.91 |
| D | 3.125 | 156.50 |
| E | 1.56250 | 42.66 |
| F | 0.78125 | 6.10 |
| G | 0 | 0.285 |
