# Supplementary material for: Cyclooxgenase-2 Inhibiting Perfluoropoly (Ethylene Glycol) Ether Theranostic Nanoemulsions—In Vitro Study
Source: PLoS One. 2013 Feb 7;8(2):e55802. doi: 10.1371/journal.pone.0055802 (PMC3567136; doi:10.1371/journal.pone.0055802)
Supplement: Table S2 — Average droplet diameter and PDI of nanoemulsions A and B before and after incubation in media. (DOC) [file pone.0055802.s014.doc]

**Table S2**

Average droplet diameter and PDI of nanoemulsions **A** and **B** before and after incubation in media.

| Media incubation | Nanoemulsion **A** | | Nanoemulsion **B** | |
| --- | --- | --- | --- | --- |
|  | Average diameter (nm) | PDI | Average diameter (nm) | PDI |
| Before | 144.6 | 0.113 | 123.8 | 0.156 |
| After | 140.6 | 0.135 | 128.7 | 0.149 |
