# Supplementary material for: Cyclooxgenase-2 Inhibiting Perfluoropoly (Ethylene Glycol) Ether Theranostic Nanoemulsions—In Vitro Study
Source: PLoS One. 2013 Feb 7;8(2):e55802. doi: 10.1371/journal.pone.0055802 (PMC3567136; doi:10.1371/journal.pone.0055802)
Supplement: Table S3 — NIR signal intensity (relative fluorescence units, RFU) of cells labeled with nanoemulsion B (A–E) and unlabeled control cells (F). Unlabeled cells were used to correct for the background fluorescence signal from the cell suspension. (DOC) [file pone.0055802.s015.doc]

**Table S3**

NIR signal intensity (relative fluorescence units, RFU) of cells labeled with nanoemulsion **B** (A-E) and unlabeled control cells (F). Unlabeled cells were used to correct for the background fluorescence signal from the cell suspension.

| Sample | Concentration of emulsion used (mg/mL) | NIR RFU |
| --- | --- | --- |
| A1 | 1.4 | 290.18 |
| A2 | 1.4 | 315.62 |
| B1 | 0.7 | 178.90 |
| B2 | 0.7 | 184.81 |
| C1 | 0.35 | 97.22 |
| C2 | 0.35 | 94.33 |
| D1 | 0.175 | 58.46 |
| D2 | 0.175 | 61.94 |
| E1 | 0.0875 | 32.55 |
| E2 | 0.0875 | 39.13 |
| F1 | 0 | 4.50 |
| F2 | 0 | 6.17 |
