# Supplementary material for: Cyclooxgenase-2 Inhibiting Perfluoropoly (Ethylene Glycol) Ether Theranostic Nanoemulsions—In Vitro Study
Source: PLoS One. 2013 Feb 7;8(2):e55802. doi: 10.1371/journal.pone.0055802 (PMC3567136; doi:10.1371/journal.pone.0055802)
Supplement: Protocol S1 — Lysosomal labeling and confocal imaging procedure. (DOC) [file pone.0055802.s016.doc]

**Lysosomal Labeling and Confocal Imaging Procedure:**

To further investigate the presence of droplets in the specific intracellular compartments, lysosomal labeling was performed. Macrophages were seeded on glass bottom confocal plates (P35G-1.0-14-C, MatTek Corporation) for 24 h at a concentration of 0.25x10^6^ cells per well. Cultured macrophages were exposed to nanoemulsion **C** for 24 h. After aspirating the medium and repeated washings with PBS, cells were exposed to Lysotracker® Green DND-26 at 150 nM concentration. After 1 h incubation, cells were washed and fixed in 4% paraformaldehyde for 30 min. Fixed cells were washed with PBS (supplemented with 1% FBS) and exposed to Hoescht dye (1µg/mL) dissolved in 1% FBS in PBS for 5 min. Cells were washed and stored in PBS at 4 ºC until imaged. Imaging was performed on Spinning Disk Confocal microscope (Andor Revolution XD) with 60x oil immersion objective (NA 1.49). The emission filters were 447/60 nm band pass filter in the 405 channel (Hoescht 33342), 794/160 nm band pass filter in the 640 channel (Cellvue® Burgundy), and 525-50/600-45/690 nm triple band pass filter in the 488 channel (Lysotracker® Green DND-26). Results are presented in Figure S11.
